# Plasticity and stringency: rethinking stem cell division modes

**DOI:** 10.1042/BST20250202

**Published:** 2026-02-02

**Authors:** Muhammed Burak Bener, Mayu Inaba

**Affiliations:** 1 Department of Cell Biology, University of Connecticut School of Medicine, Farmington, CT 06030, U.S.A.

**Keywords:** asymmetric cell division, Stem cell clonal expansion, symmetric cell division, Stem cell heterogeneity

## Abstract

Asymmetric cell division (ACD) has been extensively studied in various stem cell systems as a fundamental mechanism that ensures the balance between stem cell self-renewal and differentiation. ACD allows one daughter cell to retain stem cell identity while the other commits to differentiation, thereby maintaining tissue homeostasis over time. Stem cells also undergo symmetric cell division, in which both daughter cells adopt either stem or differentiated fates. What are the outcomes of each cell division mode, and how strictly are these modes executed across different stem cell systems? There have been technical challenges of visualizing stem cell division *in vivo* due to the structural complexity of tissues and the rarity and ambiguous identity of genuine stem cells. Despite these difficulties, recent technical advancements have revealed how these cells operate within their native environments. This review summarizes key studies that elucidate distinct division modes and their functional outcomes across various stem cell systems.

## Introduction

Stem cells are defined by their dual potential: the ability to self-renew and the capacity to differentiate into specialized cell types. This delicate balance is sustained within specialized microenvironments, termed niches, which provide the physical and molecular cues that regulate stem cell behavior [1]. Niche concepts have been extensively studied across various biological systems, including the germline stem cells (GSCs) of *Drosophila* and hematopoietic stem cells (HSCs) in mammals [2–6]. Now we have a better understanding of how stem cell fate is controlled in the stem cell niche contexts.

Mechanistically, stem cell maintenance can occur through several strategies. Asymmetric cell division (ACD) has long been considered a central mechanism, allowing a single division to produce one daughter cell that retains stem cell identity while the other differentiates. This is often achieved through stereotypical orientation of the mitotic spindle and polarized distribution of fate determinants as seen in *Drosophila* neuroblasts [7] or localized niche-derived signaling, including Janus kinases and Signal Transducers and Activators of Transcription (JAK-STAT) signaling [8–10] and Bone Morphogenetic Protein (BMP) signaling [11,12] observed in *Drosophila* gonads. By coupling polarity and signaling, ACD ensures the long-term preservation of the stem cell pool while continuously supplying differentiated progeny.

In parallel, symmetric cell division (SCD) is also important for maintenance of stem cells at the population level. Rather than each stem cell dividing asymmetrically, stem cell numbers are balanced collectively: some divisions expand the stem cell pool while others produce differentiating daughters, leading to overall homeostasis. This mode has been documented in HSCs [13,14] and intestinal stem cells [15,16]. The highly flexible nature of SCD provides plasticity to the system, thus safeguarding system resilience against stem cell loss or overproliferation.

Accumulating evidence indicates that each stem cell type can adopt different division modes but often shows a preferred one. Table 1 summarizes the current understanding of these modes across various stem cell systems. The observed proportions of ACD and SCD are further influenced by the highly dynamic and context-dependent nature of stem cell division, which can vary substantially across cell types and physiological states. Several examples have been demonstrated in which stem cells can switch between ACD and SCD, and such switching provides regulatory flexibility, allowing tissues to adapt to developmental cues, regenerative needs, and environmental changes. A remarkable example was seen in intestinal stem cells, which alter their division orientation between ACD and SCD in response to nutrient availability, thereby adjusting intestinal growth [15]. Repeated muscle injury increases symmetric renewal events in muscle stem cells, reducing clonal diversity during prolonged repair [36]. Similarly, aging promotes a bias toward symmetric divisions and disrupts epigenetic asymmetry during HSC division [37]. Inflammation also skews HSC behavior toward symmetric renewal [38]. However, the mechanism remains unclear as to how such flexible switching occurs in a context-dependent manner across many stem cell systems.

**Table 1:** Stem Cell Division Modes Across Systems

Stem cell type	Symmetric division	Asymmetric division	Method of determination	References
*Drosophila* germline stem cells	Rare (as a regenerative response to stem cell loss)	Common (steady-state)	Live imaging of spindle orientation, lineage tracing, ablation assays	[6,17–19]
*Drosophila* neuroblasts	Rare (in early development to expand the pool; or in tumorigenesis)	Common (steady-state)	Live imaging, lineage tracing, genetic perturbations	[20–23]
*Drosophila* intestinal stem cells	Common (as a response to nutrient fluctuations)	Common (steady-state)	Lineage tracing	[15,24–27]
Mouse hematopoietic stem cells	Yes (steady-state and during stress/repair)	Yes, asymmetric divisions observed under certain contexts	*In vivo* lineage tracing, paired daughter cell assays, transplantation	Reviewed in [28]
Mouse neural stem cells (Radial glia)	Symmetric early in development (expansion)	Asymmetric later (neuron vs. progenitor fate)	Lineage tracing, live imaging in brain slices	[29,30]
Mammalian intestinal stem cells (Lgr5 + crypt base columnar cells)	Predominant mode— stochastic symmetric divisions balanced at population level; ensures maintenance	Rare clear ACD events; daughters’ fates resolved via niche signals and competition	Clonal analysis using inducible multi-color reporters, clonal competition assays	[16,31,32]
Epidermal stem cells (Skin)	Yes; stochastic fate decisions at population level	Rare strict ACD	Lineage tracing	[33–35]

ACD strictly dictates distinct fates for the two daughter cells, whereas fate determination following SCD appears to occur in a more flexible manner, likely influenced by environmental cues such as niche space availability and the strength of local or systemic signals, suggesting that SCD represents an adaptive strategy to meet tissue demands. However, if ACD relies on the precise segregation of intrinsic factors at the time of division, shifting to SCD could compromise the potential outcomes. Understanding the true significance of ACD is therefore essential for interpreting the outcomes of changes in division modes.

## ACD

**Figure 1:**
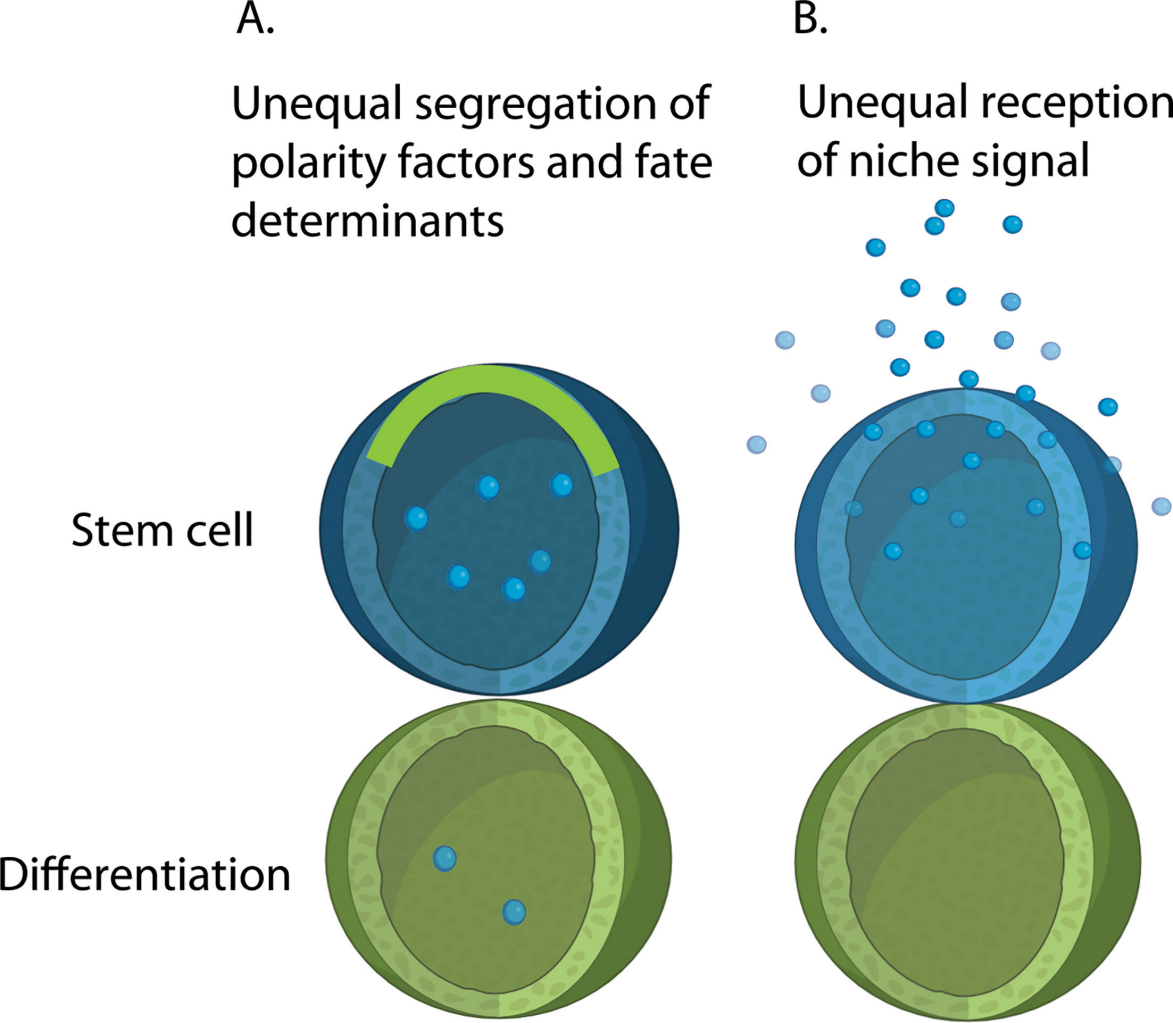
Intrinsic and extrinsic regulation of cell fates in ACD. **(A**) Intrinsic fate regulation relies on the unequal segregation of factors. Green line along the stem cell cortex indicates polarity factors (e.g. Par proteins) that later regulate asymmetric segregation of downstream effectors (e.g. Numb, Prospero, and Miranda). (**B**) In contrast, extrinsic regulation is affected by the microenvironment. The two daughter cells may be equipotent, but their subsequent fate is dictated by their proximity to the niche. The cell remaining in contact with the niche receives external signals that maintain its stemness, while the cell displaced from the niche is induced to differentiate.

The concept of ACD emerged as an elegant mechanism to explain how stem cells can simultaneously self-renew while producing differentiating progeny. In an ACD, one daughter cell inherits the molecular and positional cues necessary to maintain stemness, while the other is directed toward differentiation. The earliest mechanistic studies of ACD were conducted in *Drosophila* neuroblasts, which undergo repeated divisions during larval brain development to produce a self-renewing neuroblast and a differentiating ganglion mother cell [39]. In this case, segregation of intrinsic factors plays a central role in fate determination. At first, cell polarity proteins such as Par complex components (Bazooka/Par-3, Par-6, and aPKC) establish cortical asymmetry. Then, these cortical factors drive asymmetric segregation of key cell fate determinants, including Numb, Prospero, Miranda, and Brat, into only one daughter cell [20,40–44]. These studies provided a mechanistic blueprint for how intracellular polarity governs asymmetric outcomes (Figure 1A).

Shortly afterward, the principle of ACD was extended to GSCs in *Drosophila* ovaries and testes [6,17]. Here, niche-derived extrinsic signals are critical for fate determination. The orientation of the mitotic spindle toward the niche ensures that one daughter remains in contact with niche signals and retains stem cell identity, while the other is displaced and begins differentiation (Figure 1B). This discovery highlighted that extrinsic positional cues from the niche can work in concert with intrinsic polarity mechanisms to enforce ACD.

Since then, ACD has been recognized in multiple stem cell systems (Table 1). In mammalian neural stem cells, cortical radial glia displays asymmetric inheritance of centrosomes and fate determinants (Par proteins, Numb), producing both self-renewing progenitors and neurons [29,30]. In epithelia, stem cells can also undergo oriented divisions that determine whether progeny remain in the basal/stem cell compartment or are displaced upward to differentiate [35]. Murine HSCs are also known to exhibit evidence of ACD [45]. Similar to the case of *Drosophila* neuroblasts, asymmetric distribution of fate determinants such as Numb, Musashi, and aged mitochondria has been demonstrated (reviewed in [28]), although the extent to which ACD is required *in vivo* remains debated.

## Why ACD?

While ACD has been recognized in broad stem cell systems and prevailing theory held that ACD was essential for balancing self-renewal and differentiation, this classical model has been challenged by emerging evidence that stem cell populations can be maintained through symmetric divisions, where both daughter cells either remain as stem cells or differentiate. As long as the stem cell niche is limited, either by physical space constraints or by the availability of self-renewal signals [46], stem cells constantly compete for limited niche resources, preventing both overpopulation and depletion. This raises a fundamental question: if symmetric divisions alone can maintain stem cell populations, what is the evolutionary advantage conferred by ACD?

### 1. Asymmetric inheritance of epigenetic information

It has been shown that during ACD of fly GSCs, preexisting (‘old’) histone H3 is preferentially inherited by the self-renewing stem cell, whereas newly synthesized (‘new’) histone H3 is biased toward the differentiating daughter cell [47]. This indicates an intriguing possibility that although the DNA sequence is identical between sister chromatids, the chromatin composition (in terms of histone origin) is asymmetric, providing a potential mechanism by which identical genomes can give rise to daughter cells with distinct epigenetic states and fates. This provided the possibility that epigenetic memory (via histones) can be partitioned asymmetrically during stem-cell divisions.

### 2. Cellular aging and fate determination

**Figure 2:**
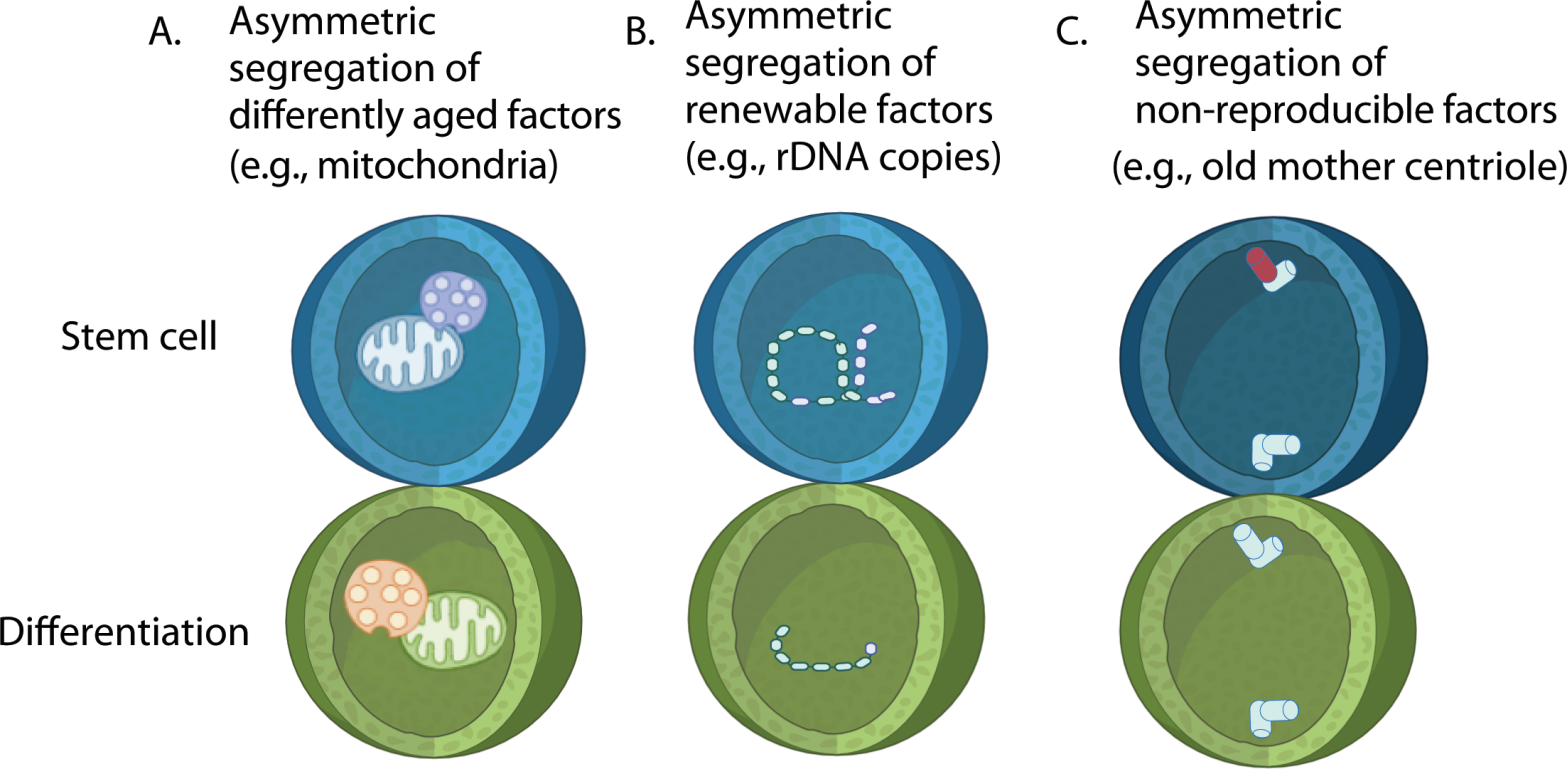
Biased segregation of factors in ACD. **(A**) Unequal segregation of differently aged proteins or cellular organelles may determine cellular ‘age’ or their cell fates. Diagram shows a lysosome and a mitochondrion. (**B**) Asymmetric segregation of renewable factors (e.g. rDNA copies). (**C**) Old mother centriole (red) is kept in *Drosophila* male GSCs over many rounds of asymmetric divisions. There has been an idea of the ‘immortal factor,’ which may be deposited in stem cells and kept for a long term throughout the lifetime of the organism. The presence of any factor associating with the old mother centriole to be kept in stem cells remains unknown.

One potential advantage of ACD lies in its role in cellular aging/rejuvenation through the unequal segregation of age-associated damage (Figure 2A). In the asymmetrically dividing yeast *Saccharomyces cerevisiae*, aging factors such as damaged protein aggregates [48,49], aged mitochondria [50], and extrachromosomal rDNA circles [51] are preferentially retained in the mother cell. This active, non-random segregation allows the daughter cell to inherit a relatively pristine cytoplasm, effectively resetting its cellular age and contributing to the yeast’s replicative lifespan.

Intriguingly, similar intrinsic asymmetries have been observed in mammalian stem cell systems. For example, mammary stem-like cells exhibit non-random mitochondrial segregation, in which older and younger mitochondria are differentially inherited by daughter cells [52]. Using a photoactivatable GFP fused to a mitochondrial outer membrane protein (paGFP-Omp25), researchers showed that labeled (older) mitochondria were preferentially inherited by the differentiating daughter cell. This asymmetric distribution generates two progenies with distinct metabolic activities, biasing them toward different fates [53].

Other organelles also show asymmetric inheritance that influences stem cell behavior. In HSCs, unequal segregation of lysosomes has been shown to determine daughter cell fate [54]. Similarly, peroxisomes display asymmetric distribution during division [55], further highlighting that the unequal inheritance of organelles and damaged components may determine the cellular age and also the strategy for fate determination across diverse stem cell systems.

### 3. rDNA maintenance across generations

Another example of ACD’s role in genome maintenance involves preserving the copy number of ribosomal DNA (rDNA) in the germline (Figure 2B). Ribosomes are responsible for translating mRNA into protein, and their core components, the ribosomal RNAs, are transcribed from rDNA loci. Because ribosome biogenesis is essential for cell survival, maintaining rDNA integrity is particularly critical for animals. However, rDNA loci are organized as highly repetitive tandem repeats, making them inherently unstable and susceptible to loss through intrachromosomal recombination and collisions between the transcription and replication machinery [51,56,57]. Indeed, gradual loss of rDNA copies has been linked to replicative aging [58,59], underscoring the importance of mechanisms that counteract rDNA loss to ensure long-term cell viability and organismal health [60].

In *Drosophila*, rDNA is located on both the X and Y chromosomes, and its deficiency causes the characteristic ‘bobbed’ phenotype, marked by short bristles and abnormal cuticles due to impaired ribosome production [61,62]. In 1968, Ferruccio Ritossa made the striking observation that flies exhibiting the bobbed phenotype could produce progeny with wildtype features, such as normal bristle size and cuticle morphology [61]. He attributed this reversion to an increase in rDNA copy number in the male germline, a process he termed ‘rDNA magnification.’

Subsequent studies confirmed that when parents with reduced rDNA copy number produce offspring, this reduction is inherited by F1 progeny but gradually restored within approximately 10 days [63]. This recovery parallels the phenotypic reversion seen in bobbed flies, suggesting that rDNA copy number is actively maintained across generations. One of the leading models explaining this phenomenon is unequal sister chromatid exchange (USCE) [64]. USCE is triggered when rDNA copy number falls below a critical threshold. In GSCs undergoing rDNA magnification, transcription of retrotransposon R2 is markedly up-regulated, generating double-stranded DNA breaks in the male GSCs [65]. These breaks promote recombination between sister chromatids, allowing one chromatid to gain rDNA copies at the expense of the other, thereby increasing rDNA content in one of the sister chromatids.

During ACD of male GSCs, sister chromatids of the X and Y chromosomes segregate non-randomly [66]. As a result, the stem cell preferentially inherits the chromatid with the higher rDNA copy number, thereby preserving and amplifying rDNA within the GSC lineage. Although the USCE model initially faced limitations, such that observed rDNA recovery often exceeded the doubling expected from a single exchange event, recent research has clarified this discrepancy. A mathematical model demonstrated that repeated rounds of ACD can account for the greater-than-doubling increase in rDNA copy number observed experimentally [67]. Because GSCs can undergo multiple asymmetric divisions before differentiating, these successive ACDs enable cumulative amplification of rDNA. Thus, ACD likely serves as a crucial mechanism coupling USCE with selective chromatid inheritance to drive rDNA magnification and preserve rDNA integrity across generations.

### 4. Centrosome asymmetry

Centrosome asymmetry contributes to stem cell fate by influencing how asymmetric cell divisions are executed [68]. Because the two centrosomes in a cell differ in age and microtubule-organizing activity, they can differentially affect cell polarity and spindle orientation, the key features of asymmetric division. In *Drosophila* male GSCs, the old mother centriole is maintained over many cell division cycles [69]. Interestingly, this asymmetry of the centriole age/size influences the segregation pattern of many other cellular factors in dividing daughter cells [70], raising the possibility that centrosomes act not only as polarity organizers but also as carriers of fate-influencing information. Other than *Drosophila* male GSCs, many stem cells preferentially inherit either the older mother centrosome or the younger daughter centrosome (reviewed in [68]), reflecting the conserved nature of the centrosome function, which also supports this idea. It remains unclear whether centrosome age (mother vs. daughter) determines their association with fate determinants or whether an ‘immortal factor’ resides on the old mother centriole and persists in stem cells throughout the organism’s lifetime.

### The role of ACD on clonal expansion

**Figure 3:**
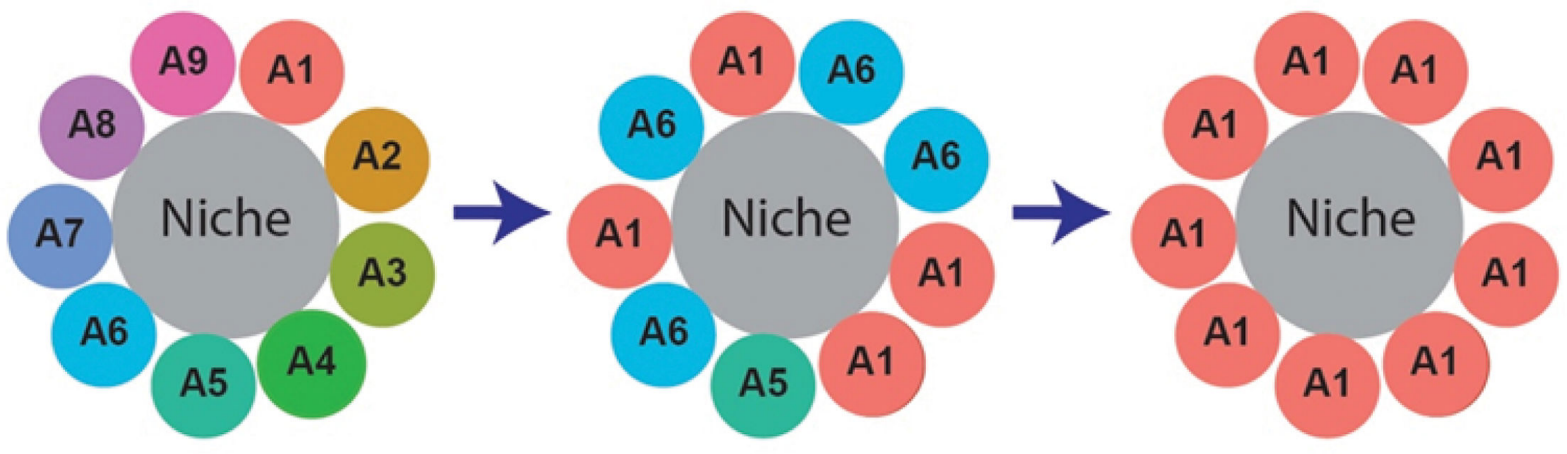
The process of clonal expansion via neutral drift. A cartoon illustrating the process of clonal expansion driven by neutral drift within a stem cell niche over time. The niche is initially occupied by numerous distinct stem cell clones (A1-A9, represented by different colors, left panel). Over time, neutral competition leads to the random expansion and contraction of individual clones (middle panel). This process ultimately results in clonal dominance, where the niche becomes monoclonal (right panel), populated entirely by the descendants of a single stem cell clone (**A1**).

In a stem cell pool, any deviation from strict ACD, through symmetric divisions, dedifferentiation, or stem cell loss, inevitably reduces clonal diversity [71] (Figure 3). This process, known as neutral drift, results in the random expansion and contraction of individual stem cell clones, eventually leading to the dominance of a single clone within the stem cell pool [16,31].

The mammalian intestinal epithelium provides a compelling example of clonal expansion. Intestinal stem cells reside within millions of tiny, compartmentalized structures called crypts. Clonal dynamics of intestinal stem cells have been investigated using the Cre-loxP system coupled with a Confetti reporter cassette [16,31]. This system uses Cre recombinase to randomly activate one of four fluorescent proteins within cells, enabling visualization and tracking of individual stem cells and their progeny by their distinct colors. These studies revealed that the entire crypt becomes populated by the descendants of a single stem cell clone in ~3 months post induction of marking, thus becoming monoclonal [16,31]. Similar clonal dynamics have been observed in the spermatogonial stem cells of the mammalian testis [72] and are now recognized as a common feature of many tissues, including skin, liver, and esophagus [73,74].

The clinical consequence of clonal expansion is evident in the human hematopoietic system through a phenomenon known as clonal hematopoiesis (CH) [75–77]. This age-related expansion of HSC clones becomes increasingly prevalent with age and is linked to an increased risk of cardiovascular disease and hematological malignancies [75,76,78]. Advances in next-generation sequencing and single-cell technologies have enabled high-resolution lineage tracing by detecting naturally occurring cellular barcodes, such as somatic mitochondrial DNA mutations or unique methylation patterns [79,80]. This makes it possible to infer clonal expansion even without known driver genes. Several mathematical modeling studies in hematopoiesis have provided insights into the mechanisms driving the clonal expansion, demonstrating that smaller stem cell pools [81] and increased proliferation rates [82] accelerate this process. Despite the widespread observation of clonal expansion in nearly all tissues and its significant clinical implications, as evidenced by CH, the precise mechanisms driving clonal expansion remain incompletely understood.

Our recent study using the *Drosophila* testis as a model system provided mechanistic insight into how stem cell division modes regulate clonal dynamics [83]. We first developed a mathematical model predicting that a higher ACD rate significantly delays the time required for a single clone to dominate the niche. Then, using *in vivo* lineage tracing, we confirmed that under physiological conditions, GSC clones undergo neutral competition, which leads to their elimination or expansion within the niche (Figure 3). These findings provide direct evidence that ACD is a key mechanism for suppressing clonal dominance and maintaining stem cell heterogeneity within the niche.

## SCD

SCD plays a crucial role in maintaining stem cells at the population level. Instead of each stem cell being restricted to divide asymmetrically, SCD allows stem cell numbers to be regulated through the collective behavior of the entire stem cell population. In this model, some stem cells undergo symmetric self-renewal, producing two stem cell daughters and thereby expanding the pool, while others undergo symmetric differentiation, generating two committed progenitors that contribute to tissue formation. The balance between these two symmetric outcomes ensures overall population homeostasis, even though individual stem cells may fluctuate in their division mode.

This population-level strategy has been proposed and explained in several systems, including HSCs [13,14], where stochastic switches between symmetric self-renewal and differentiation maintain long-term hematopoietic balance, and intestinal stem cells [15,16], where dynamic competition and neutral drift among stem cells lead to population replacement without loss of tissue integrity. Similar mechanisms have also been reported in other rapidly renewing tissues such as the epidermis and germline, highlighting the generality of this principle across different organisms and tissue contexts (Table 1). Because of the flexible and adaptive nature of SCD, it can provide plasticity to stem cell systems. By allowing reversible changes in the proportion of self-renewing vs. differentiating divisions, tissues can rapidly adjust to physiological demands.

## Dedifferentiation and transdifferentiation

A third mechanism contributing to stem cell homeostasis is dedifferentiation, in which more committed progenitor or differentiated cells revert to a stem-like state. Similar to the role of SCD, dedifferentiation enhances the plasticity and resilience of stem cell systems, allowing tissues to adapt to environmental or physiological changes such as starvation or stress conditions that reduce stem cell numbers [84]. Accumulating evidence supports the occurrence of dedifferentiation across diverse organisms and tissues (Table 2). In *Drosophila* testes, lost GSCs can be replenished by dedifferentiation of spermatogonia [84,85,113], while in mammals, similar processes have been observed during spermatogenesis [93,94], in neural progenitors [111], and in epithelial cells [109], which can revert to a more primitive state under certain conditions. A recent study in melanocyte stem cells further suggests that cells in the transit-amplifying region can frequently revert to the stem cell state [92] . However, in most cases, dedifferentiation appears to be a rare event, activated only under specific physiological or stress-induced contexts.

**Table 2:** Stem Cell Dedifferentiation Across Systems

Stem cell system	Source of dedifferentiation	Detection method	References
*Drosophila* male germline stem cells	Gonialblasts or spermatogonia	Live imaging, lineage tracing, ablation assays	[18,84–89]
*Drosophila* female germline stem cells	Cystoblasts	Lineage tracing, ablation assays	[90,91]
Melanocyte stem cells	Transit amplifying cells	Live imaging, single-cell transcriptome analysis	[92]
Mouse germline stem cells	GFRA1^+^ and NGN3^+^ spermatogonia (A_pr_ and A_al_) KIT^+^ spermatogonia (A_1_)	Live imaging, lineage tracing, ablation assays, transplantation assays	[72,93–96]
*Drosophila* intestinal stem cells	Enteroblasts	Lineage tracing, ablation assays	[97]
Mammalian intestinal stem cells	Various progenitor and differentiated cell types (e.g. Dll1^+^ secretory progenitor cells, Alpi^+^ enterocytes, Bmi1^+^ enteroendocrine cells, Tuft cells, Goblet cell progenitors, Paneth cells)	Lineage tracing, ablation assays, chemical damage induction (bleomycin, dextran sodium sulfate)	[98–108]
Mouse airway stem cells	Secretory cells	Lineage tracing, ablation assays	[109]
Mouse pancreas (β-cells)	α- or δ-cells can convert into β-like cells after β-cell loss	Genetic lineage tracing, cell ablation, regeneration models reported as ‘transdifferentiation.’	[110]
Mouse neural stem cells	Astrocytes	Lineage tracing	[111,112]

It remains controversial whether dedifferentiation is essential for maintaining stem cell numbers under steady-state conditions. One major complication arises from how dedifferentiation is defined and experimentally determined, which varies substantially across systems. Some studies identify dedifferentiation based on molecular marker conversion, for instance, re-expression of stemness-associated transcription factors in differentiated cells, while others rely on cellular relocation relative to the niche, assuming that re-entry into the niche environment reflects a return to stemness. This definitional ambiguity often leads to discrepancies in interpreting data and comparing results among different tissues or model organisms.

In mouse spermatogenesis, researchers often describe this process using the terms ‘reversion’ or ‘interconversion’ rather than ‘dedifferentiation,’ emphasizing the dynamic exchange between undifferentiated and differentiating spermatogonia [93,94]. By contrast, in the *Drosophila* GSC system, once a stem cell daughter leaves the niche, it is fated for differentiation. Because the ACD mode is predominant and the orientation of the division is well established, cellular position relative to the niche is the most reliable criterion for distinguishing stem vs. differentiating cells, and dedifferentiation is defined primarily by a physical relocation back into the niche [17]. In this system, a recent study revealed that dedifferentiation occurs even under normal physiological conditions [89], and regeneration assays further showed that it serves as a major mechanism for replenishing lost stem cells [89]. Such behavior seen in this system is reminiscent of mouse spermatogonia [93,94], and melanocyte stem cells [92].

Given that the cellular machinery governing ACD is tightly regulated in many systems, dedifferentiation may provide an alternative strategy for restoring niche occupancy rather than shifting from ACD to SCD. The molecular mechanism remains largely unknown how cells sense niche vacancy and initiate dedifferentiation.

Together, these studies highlight that the conceptual and operational definitions of dedifferentiation remain fluid, and this ambiguity complicates efforts to determine its frequency, regulatory mechanisms, and physiological relevance across systems. A standardized framework for identifying dedifferentiation, combining molecular, spatial, and functional criteria, will be essential for resolving these discrepancies and clarifying its contribution to tissue homeostasis.

## Discussion

Despite the structural and molecular complexity of stem cell niches, recent studies have begun to reveal that various types of stem cells can switch between different division modes, ACD and SCD. These findings indicate that ACD and SCD are not fixed properties of particular stem cell types but rather flexible strategies that allow stem cells to maintain both robustness and adaptability within their tissue contexts. Moreover, these observations suggest the existence of regulatory mechanisms that dynamically govern the balance between these division modes. The mechanism by which stem cells sense the tissue demand and align it to their division mode remains largely unknown.

ACD has been documented in a wide range of mammalian stem cell systems [53,54,114,115]. Conversely, several mammalian stem cell populations, such as those in the intestine, are often described as being maintained predominantly through symmetric divisions [16,31,46,116]. However, the wide variation in reported ACD frequencies even within the same tissue types [117] suggests that current methodologies may still be challenging to capture the true distribution of division modes. These discrepancies arise partly from the technical difficulty of directly visualizing and classifying division outcomes *in vivo*.

ACD can profoundly influence long-term clonal dynamics by slowing clonal expansion and preserving heterogeneity. Thus, re-evaluating the prevalence and functional significance of ACD using advanced imaging and quantitative lineage-tracing approaches will be essential to achieve a comprehensive understanding of stem cell population dynamics. Importantly, modulating the mode of stem cell division, particularly enhancing ACD, could represent a promising disease prevention strategy to minimize clonal expansion and mitigate the onset of age-associated diseases such as CH.

**Perspectives**

- **Highlight the importance of the field.** Stem cell maintenance is governed by multiple strategies, including asymmetric cell division (ACD), symmetric cell division (SCD), and dedifferentiation. Relative contributions of them vary by system, context, and age. The tight regulation of these ‘modes’ is important to understand how stem cell systems adapt environmental changes.
- **Provide a summary of the current thinking.** Although alternative strategies to ACD provide flexibility, ensuring resilience under stress or niche perturbation, loss of ACD fidelity or shifts toward SCD or dedifferentiation can have profound consequences, from tissue dysfunction to cancer initiation. This review mainly highlights these potential consequences.
- **Comment on future directions.** Understanding how these division modes are integrated, modulated by competition, and influenced by aging remains a central challenge. Elucidating molecular determinants of division choice, developing therapies to prevent clonal expansion, and leveraging dedifferentiation for regenerative medicine. By integrating insights across systems, we can begin to define principles that safeguard stem cell function throughout life and inform strategies to treat stem cell–related diseases.

## Open Access

Open access for this article was enabled by the participation of University of Connecticut in an all-inclusive Read & Publish agreement with Portland Press and the Biochemical Society.

## Abbreviations

ACD: asymmetric cell division
CH: clonal hematopoiesis
GSC: germline stem cell
GSCs: germline stem cells
HSC: hematopoietic stem cell
HSCs: hematopoietic stem cells
SCD: symmetric cell division
USCE: unequal sister chromatid exchange
rDNA: ribosomal DNA
